# Involvement of Peptidoglycan Receptor Proteins in Mediating the Growth-Promoting Effects of *Bacillus pumilus* TUAT1 in *Arabidopsis thaliana*

**DOI:** 10.1093/pcp/pcae016

**Published:** 2024-02-19

**Authors:** Md. Monirul Islam, Shin-ichiro Agake, Takehiro Ito, Safiullah Habibi, Michiko Yasuda, Tetsuya Yamada, Gary Stacey, Naoko Ohkama-Ohtsu

**Affiliations:** Plant Biotechnology and Genetic Engineering Division, Institute of Food and Radiation Biology, Bangladesh Atomic Energy Commission, Dhaka 1207, Bangladesh; United Graduate School of Agriculture, Tokyo University of Agriculture and Technology, 3-5-8 Saiwaicho, Fuchu-shi, Tokyo, 183-8509 Japan; Institute of Global Innovation Research, Tokyo University of Agriculture and Technology, 3-8-1 Harumicho, Fuchu-shi, Tokyo, 183-8538 Japan; United Graduate School of Agriculture, Tokyo University of Agriculture and Technology, 3-5-8 Saiwaicho, Fuchu-shi, Tokyo, 183-8509 Japan; Institute of Agriculture, Tokyo University of Agriculture and Technology, 3-5-8 Saiwaicho, Fuchu-shi, Tokyo, 183-8509 Japan; Institute of Global Innovation Research, Tokyo University of Agriculture and Technology, 3-8-1 Harumicho, Fuchu-shi, Tokyo, 183-8538 Japan; Institute of Agriculture, Tokyo University of Agriculture and Technology, 3-5-8 Saiwaicho, Fuchu-shi, Tokyo, 183-8509 Japan; Institute of Global Innovation Research, Tokyo University of Agriculture and Technology, 3-8-1 Harumicho, Fuchu-shi, Tokyo, 183-8538 Japan; Institute of Agriculture, Tokyo University of Agriculture and Technology, 3-5-8 Saiwaicho, Fuchu-shi, Tokyo, 183-8509 Japan; Institute of Global Innovation Research, Tokyo University of Agriculture and Technology, 3-8-1 Harumicho, Fuchu-shi, Tokyo, 183-8538 Japan; Division of Plant Science and Technology, University of Missouri-Columbia—Bond Life Science Center, 1201 Rollins St., Columbia, MO 65201-4231, USA; Institute of Global Innovation Research, Tokyo University of Agriculture and Technology, 3-8-1 Harumicho, Fuchu-shi, Tokyo, 183-8538 Japan; Institute of Agriculture, Tokyo University of Agriculture and Technology, 3-5-8 Saiwaicho, Fuchu-shi, Tokyo, 183-8509 Japan

**Keywords:** *Bacillus pumilus* TUAT1, Microbe-associated molecular patterns, Pattern recognition receptors, Peptidoglycans, RNA-seq

## Abstract

*Bacillus pumilus* TUAT1 acts as plant growth–promoting rhizobacteria for various plants like rice and *Arabidopsis*. Under stress conditions, *B. pumilus* TUAT1 forms spores with a thick peptidoglycan (PGN) cell wall. Previous research showed that spores were significantly more effective than vegetative cells in enhancing plant growth. In *Arabidopsis*, lysin motif proteins, LYM1, LYM3 and CERK1, are required for recognizing bacterial PGNs to mediate immunity. Here, we examined the involvement of PGN receptor proteins in the plant growth promotion (PGP) effects of *B. pumilus* TUAT1 using *Arabidopsis* mutants defective in PGN receptors. Root growth of wild-type (WT), *cerk1-1, lym1-1* and *lym1-2* mutant plants was significantly increased by TUAT1 inoculation, but this was not the case for *lym3-1* and *lym3-2* mutant plants. RNA-seq analysis revealed that the expression of a number of defense-related genes was upregulated in *lym3* mutant plants. These results suggested that *B. pumilus* TUAT1 may act to reduce the defense response, which is dependent on a functional LYM3. The expression of the defense-responsive gene, *WRKY29*, was significantly induced by the elicitor flg-22, in both WT and *lym3* mutant plants, while this induction was significantly reduced by treatment with *B. pumilus* TUAT1 and PGNs in WT, but not in *lym3* mutant plants. These findings suggest that the PGNs of *B. pumilus* TUAT1 may be recognized by the LYM3 receptor protein, suppressing the defense response, which results in plant growth promotion in a trade-off between defense and growth.

## Introduction

In plants, pattern recognition receptors (PRRs) recognize elicitors, such as microbe-associated molecular patterns (MAMPs), leading to MAMP-triggered immunity. Many bacterial elicitors act as MAMPs, such as flagellin, elongation factor Tu, lipopolysaccharides and peptidoglycans (PGNs), which activate plant defense responses (Newman et al. 2013). PRRs are located on plant cell membranes and have different receptor domains, such as extracellular ligand-binding, transmembrane, and intracellular kinase domains. In *Arabidopsis*, lysin motif (LysM)–containing receptor proteins, LYM1 and LYM3, act as extracellular ligand-binding domains that can physically interact with PGNs in conjunction with the LysM-containing receptor kinase, CERK1, initiating an intracellular signal transduction cascade to increase plant immunity (Willmann et al. 2011). PGNs compose the bacterial cell wall and consist of a glycan backbone of alternating β-1,4-linked N-acetylglucosamine (GlcNAc) and N-acetylmuramic acid (Glauner et al. 1988). The polymeric carbohydrate backbone of PGNs, GlcNAc, is recognized by plant LysM receptors in plants. Plant LysM domain proteins have been widely implicated in the recognition of GlcNAc-containing glycans such as Nod factors (Limpens et al. 2003, Radutoiu et al. 2003) and chitin (Kaku et al. 2006, Miya et al. 2007, Wan et al. 2008, Shimizu et al. 2010). PGNs are common MAMPs that elicit defense responses through recognition by receptor proteins in *Arabidopsis* (Gust et al. 2007, Erbs et al. 2008, Millet et al. 2010). In contrast, another MAMP, flagellin, is recognized by FLAGELLIN-SENSING 2 which is a receptor-like kinase (RLK) protein consisting of an extracellular leucine-rich repeat, a single membrane-spanning domain and a cytoplasmic serine/threonine kinase domain. Flagellin releases a conserved 22-amino acid peptide, flg-22, and triggered immune responses such as callose deposition, reactive oxygen species (ROS) production and changes in defense gene expression in *Arabidopsis*. However, this reaction can be suppressed by Nod factor and chitotetraose. Mutant (*lyk3*) studies suggested that the AtLYK3, a LysM-containing receptor kinase, is necessary for Nod factor suppression of immunity triggered by flg-22 (Liang et al. 2013).

*Bacillus pumilus* TUAT1 was originally isolated from cultivated soil taken from the fields adjacent to the Tokyo University of Agriculture and Technology (Djedidi et al. 2014). Subsequently, this strain was shown to act as a plant growth–promoting rhizobacterium (PGPR), contributing to the improvement of plant growth by inoculation on various plants, such as rice, *Setaria*, soybean (Win et al. 2018, 2019, 2020, Agake et al. 2021, 2022a, 2022b, Hasibuan et al. 2021) and *Arabidopsis* (Borjigin et al. 2024). It is now commercially available as a biofertilizer ‘Yume-bio’ (Asahi Agria. Co., Ltd. Tokyo, Japan). Under stress conditions, *B. pumilus* TUAT1 forms spores, which makes this a convenient strain for long-term storage and packaging for use as a biofertilizer. Previous research compared the PGP effects of spores and vegetative cells and found that the former were significant more effective (Ngo et al. 2019). The autoclaved dead spores of *Bacillus* spp. accelerated the growth of rice seedlings (Seerat et al. 2019), suggesting that at least one substance responsible for the PGP effects is present in heat-treated spores. The cell wall of Gram-positive bacteria, including *Bacillus*, consists of thick PGN layers, and the amounts of PGN are notably higher in spores. This has led to the hypothesis that PGN is one of the candidates inducing the PGP effects of *B. pumilus* TUAT1.

However, despite numerous reports on the effects by *Bacillus* PGPR strains, the mechanisms by which plant growth is promoted by these strains remain an open question. Additionally, it is important to clarify this mechanism to further improve the effectiveness of the *B. pumilus* TUAT1 strain as a commercial biofertilizer. The first step in this PGP process is perception of *B. pumilus* TUAT1 strain by plants, which initiates a signaling cascade leading to growth promotion. To gain insights into this, we investigated the involvement of receptor proteins for pathogen PGNs, i.e. LYM1, LYM3 and CERK1, in the perception of *B. pumilus* TUAT1 using *Arabidopsis* T-DNA knockout mutant plants. The plant responses to purified PGNs of *B. pumilus* TUAT1 were also investigated.

## Results

### Growth promotion effects of B. pumilus TUAT1 spores in the *lym1*, *lym3* and *cerk1* mutants

**Fig. 1:**
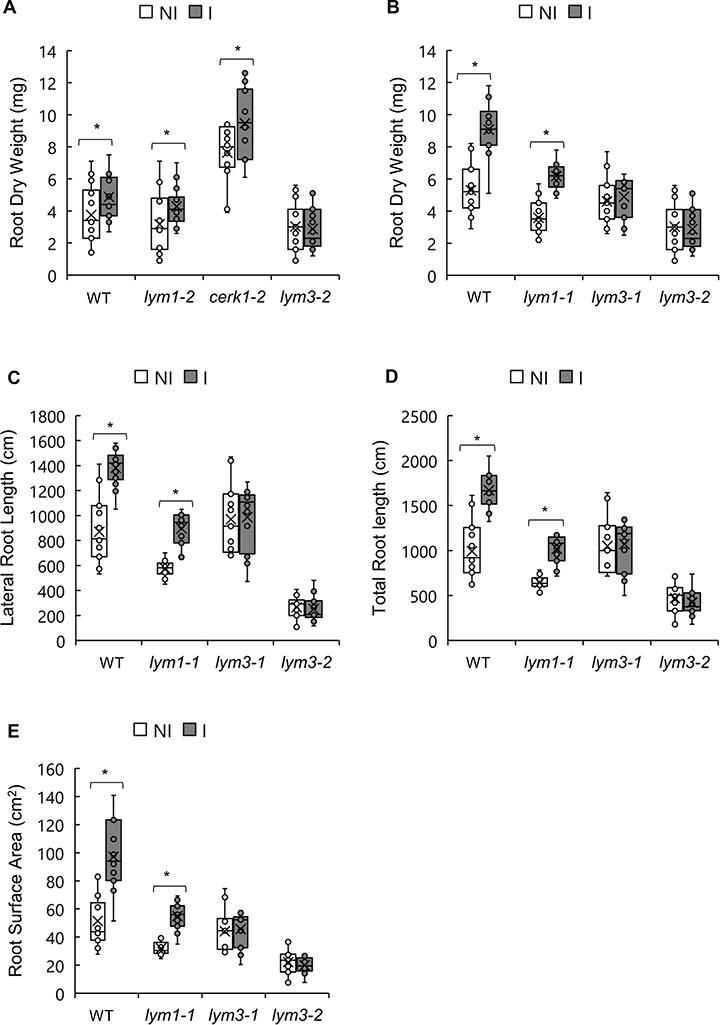
Growth promotion effects of *B. pumilus* TUAT1 in *lym1*, *lym3* and *cerk1* mutants. Root dry weights in the first (A) and second (B) experiments; lateral root length (C), total root length (D) and root surface area (E) in the second experiment. NI, not inoculated; I, inoculated with *B. pumilus* TUAT1. Boxplots denote the span from 25th to the 75th percentile and are centered on the median data of 15 biological replicates. Asterisks indicate significant differences between I and NI for each genotype by Student’s *t*-test (*P* ≤ 0.05). *n* = 15.

To determine the involvement of PGN receptors in growth promotion by *B. pumilus* TUAT1 spores in *Arabidopsis*, knockout mutant plants for *lym1-1, lym1-2, lym3-1, lym3-2* and *cerk1-2* with T-DNAs in the homozygous state of the *LYM1, LYM3* and *CERK1* genes, respectively, were established. All these mutants are described in Willmann et al. (2011). First, one mutant per each gene *lym1-2, lym3-2* or *cerk1-2* was analyzed along with the corresponding wild-type (WT) Col-0 plants. As *B. pumilus* TUAT1 is known to promote root growth (Ngo et al. 2019, Agake et al. 2021, 2022a, 2022b), we focused on roots. A significant increase in root dry weight by *B. pumilus* TUAT1 inoculation was observed in WT, *lym1-2* and *cerk1-2* plants, whereas *lym3-2* mutant plants showed no increase in root weight (**Fig. 1A**).

To confirm the different responses in *lym3* mutant from those in WT plants, another allelic mutant, *lym3-1*, was analyzed together with *lym3-2. lym1-1* was also included to confirm the result of *lym1-2*. Significant increases in root dry weight, lateral and total root lengths and root surface area by *B. pumilus* TUAT1 inoculation were observed in WT and *lym1-1* plants, but similar changes were not seen with *lym3-1* and *lym3-2* plants (**Fig. 1B–E**). These results clearly implicate the LYM3 receptor protein in mediating the PGP effects of the *B. pumilus* TUAT1 spores.

### Growth promotion effects of purified PGNs from *B. pumilus* TUAT1

**Fig. 2:**
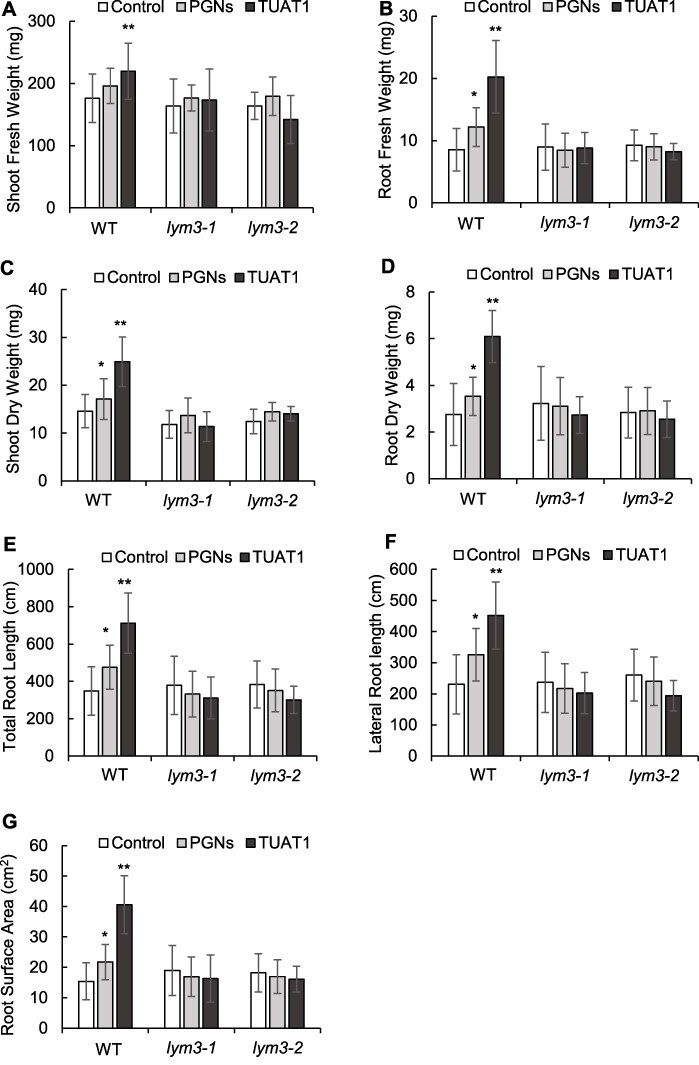
Growth promotion effects of PGNs purified from *B. pumilus* TUAT1 in *lym3* mutants. Fresh and dry weights of shoots and roots (A–D), total root length (E) and lateral root length (F). Control, treated with water; PGNs, treated with purified PGNs from *B. pumilus* TUAT1; TUAT1, treated with viable spores of *B. pumilus* TUAT1. The error bars indicate standard errors. Asterisks * and ** indicate significant difference from the control in each genotype by Student’s *t*-test at *P* ≤ 0.05 and 0.01 levels, respectively. *n* = 15.

Since LYM3, one of PGN receptors, was found to be involved in the response to the spores of *B. pumilus* TUAT1, we next confirmed if PGNs purified from this strain promote plant growth. WT, *lym3-1* and *lym3-2* plants were treated with purified PGNs from *B. pumilus* TUAT1 or spores of the strain (**Fig. 2**). Significant growth promotion was observed in WT by treatments of purified PGNs or spores, but not in either *lym3-1* or *lym3-2* plant. These results indicate that PGNs themselves have growth promotion activity and LYM3 is involved in the mechanism.

### Transcriptome analysis by inoculation of *B. pumilus* TUAT1 in *Arabidopsis* roots

**Fig. 3:**
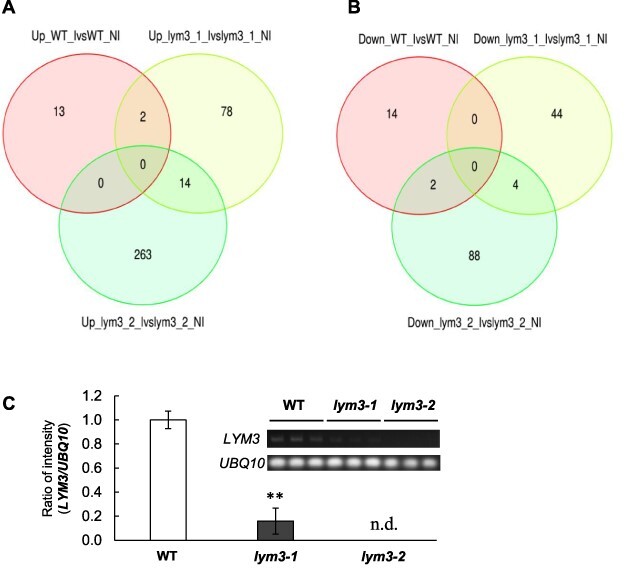
DEGs by *B. pumilus* TUAT1 treatment in roots of WT, *lym3-1* and *lym3-2* plants and quantification of LYM3 transcripts. Venn diagram of differentially upregulated genes (A) and downregulated genes (B) in WT, *lym3-1* and *lym3-2*. NI, not inoculated; I, inoculated with *B. pumilus* TUAT1. Quantification of *LYM3* transcripts (C). Data are shown as the mean ± SD from three independent biological replicate as the value of WT as 1. Asterisks indicate a significant difference from the controls by Dunnett’s test (*P* ≤ 0.01). The images of gel of the bands of *LYM3* and *UBQ10* transcripts by RT-PCR are also shown.

To further explore the plant response to inoculation with *B. pumilus* TUAT1 spores, RNA-seq analysis was used to compare uninoculated and inoculated WT, *lym3-1* and *lym3-2* plants. Total RNA was extracted from roots treated with *B. pumilus* TUAT1 spores for 1 h and subjected to transcriptome analysis. This time point, 1 hour, was chosen based on our previous reports (Xiao et al. 2023) in which of the gene expression response at 1 h after *B. pumilus* TUAT1 inoculation in rice seeds correlated with growth promotion. An average of 40.1 million clean reads mapped to the *Arabidopsis* genome at an average rate of 95.2% (**Supplementary Table S1**). As shown in the Venn diagram (**Fig. 3**) and the volcano plots (**Supplementary Fig. S1A–C**), the numbers of differentially expressed genes (DEGs) are much higher in *lym3-1* and *lym3-2*, compared to those in WT plants. Principal component analysis (PCA) shows that distribution of DEGs of two allelic mutants is not close (**Supplementary Fig. S1D**), corresponding to the small overlap between *lym3-1* and *lym3-2* in the Venn diagram (**Fig. 3**). Additionally, the dendrogram of transcriptome data indicates clear separation among all genotypes (**Supplementary Fig. S1E**). Top 1,000 genes in the expression level were clustered into eight clusters in terms of their pathways using the *K*-means method (**Supplementary Fig. S2A**). Among them, clusters 2 and 7 showed similar patterns in *lym3-1* and *lym3-2*, which are different from WT plants. Genes in cluster 2 were upregulated by inoculation of *B. pumilus* TUAT1 in WT, while they were downregulated in *lym3-1* and *lym3-2* (**Supplementary Tables S4** and **S6**). In contrast, genes in cluster 7 were downregulated by inoculation of *B. pumilus* TUAT1 in WT, while they were upregulated in *lym3-1* and *lym3-2* (**Supplementary Tables S5** and **S7**).

For the DEGs relevant to the *LYM3* mutation, common DEGs observed in both *lym3-1* and *lym3-2* were selected. For the DEGs of WT, the DEGs observed in WT and only one allele of two *lym3* mutants were included as they are irrelevant of *LYM3* mutation. Thus, the numbers of upregulated and downregulated DEGs of WT are 15 and 16, and those observed in both alleles of *lym3*, which are relevant to *LYM3* mutation, are 14 and 4, respectively (**Fig. 3**).

The locus tags and gene descriptions for the 31 DEGs of WT are presented in **Table 1**, and those for the 18 DEGs commonly observed in both *lym3* alleles are presented in **Table 2**. These 18 genes are possible candidates related to the different responses to *B. pumilus* TUAT1 in the *lym3* mutant compared to WT plants due to the lack of LYM3 protein.

**Table 1:** DEGs in WT plants by *B. pumilus* TUAT1 inoculation (*Q* ≤ 0.05)

Locus tag	Gene description	Log_2_ FC in WT	FDR value	Regulation
AT1G21520	Uncharacterized protein	0.83	0.025	Up
AT1G24881	F-box family protein	0.84	0.013	Up
AT1G26240	Proline-rich extensin-like family protein	0.85	0.001	Up
AT1G29100	Heavy metal transport/detoxification superfamily protein	0.97	0.003	Up
AT1G62280	SLAH1, SLAC1 homolog 1	0.72	0.025	Up
AT1G63570*^a^*	Receptor-like protein kinase-related family protein	1.01	0.024	Up
AT1G69490	NAP, NAC-like, activated by AP3/PI	0.63	0.025	Up
AT3G46230	HSP17.4, heat shock protein	1.39	0.007	Up
AT4G00910	Aluminum-activated malate transporter family protein	0.62	0.020	Up
AT4G08380^a^	Proline-rich extensin-like family protein	0.98	0.002	Up
AT4G10540	Subtilase family protein	1.83	3.3E-10	Up
AT4G20070	AAH, allantoate amidohydrolase	0.59	0.012	Up
AT4G38340	Plant regulator RWP-RK family protein	1.05	0.025	Up
AT5G38910	RmlC-like cupins superfamily protein	0.95	0.013	Up
AT5G40010	AATP1, AAA-ATPase 1	0.85	0.026	Up
AT1G17710	PEPC1, pyridoxal phosphate phosphatase-related protein	−2.32	6.0E-09	Down
AT1G68740	PHO1; H1 EXS (ERD1/XPR1/SYG1) family protein	−1.07	0.025	Down
AT1G73010	PS2, inorganic pyrophosphatase 1	−1.08	0.001	Down
AT2G34210	Transcription elongation factor Spt5	−2.99	3.3E-04	Down
AT2G43610^a^	Chitinase family protein	−0.67	0.012	Down
AT2G44370	Cysteine/histidine-rich C1 domain family protein	−0.83	0.007	Down
AT3G09922	IPS1, induced by phosphate starvation 1	−1.49	2.7E-06	Down
AT3G10720^a^	Plant invertase/pectin methylesterase inhibitor superfamily	−0.63	0.030	Down
AT3G25790	myb-like transcription factor family protein	−1.26	0.001	Down
AT4G12545	Bifunctional inhibitor 2S albumin superfamily protein	−0.64	0.025	Down
AT4G25160	U-box domain-containing protein kinase family protein	−0.88	0.012	Down
AT5G09980	PROPEP4, elicitor peptide 4 precursor	−0.93	0.040	Down
AT5G14330	Transmembrane protein	−0.65	0.013	Down
AT5G17340	Putative membrane lipoprotein	−1.82	2.1E-06	Down
AT5G20150	SPX1, SPX domain–containing protein 1	−1.97	5.5E-06	Down
AT5G20790	Uncharacterized protein	−1.28	0.021	Down

Gene descriptions are according to The National Center for Biotechnology Information (https://www.ncbi.nlm.nih.gov/).

a Asterisks at locus tag are DEGs detected WT and one of two *lym3* allele.

**Table 2:** Common DEGs in *lym3-1* and *lym3-2* plants by *B. pumilus* TUAT1 inoculation (*Q* ≤ 0.05)

Locus tag	Gene description*^a^*	Log_2_ FC *lym3-1*	FDR value	log_2_ FC *lym3-2*	FDR value	Regulation
AT1G05010	EFE, ethylene-forming enzyme	0.61	0.040	0.63	0.002	Up
AT1G07620	ATOBGM, GTP-binding protein Obg/CgtA	1.28	1.3E-04	0.81	0.001	Up
AT1G48490	Protein kinase superfamily protein	0.56	0.044	0.46	0.004	Up
AT1G70850	MLP34, MLP-like protein 34	0.71	0.005	0.70	0.007	Up
AT1G72150	PATL1, PATELLIN 1	0.68	3.9E-04	0.43	0.002	Up
AT1G74590	GSTU10, glutathione S-transferase TAU 10	1.02	0.033	0.91	0.030	Up
AT2G01520	MLP328, MLP-like protein 328	0.56	0.027	0.61	0.028	Up
AT2G01530	MLP329, MLP-like protein 329	0.57	0.034	0.45	0.032	Up
AT2G23030	SNRK2.9, SNF1-related protein kinase 2.9	0.57	0.041	0.51	0.011	Up
AT3G01290	HIR2, SPFH domain–containing membrane-associated protein family	0.59	0.013	0.38	0.047	Up
AT3G13750	BGAL1, beta galactosidase 1	0.75	1.5E-05	0.53	0.004	Up
AT4G07820	CAP (cysteine-rich secretory proteins, Antigen 5, and pathogenesis-related 1 protein) superfamily protein	0.96	0.032	0.45	0.045	Up
AT4G20260	PCAP1, plasma membrane–associated cation-binding protein 1	0.48	0.041	0.42	0.002	Up
AT4G34920	PLC-like phospho-diesterases Superfamily protein	0.51	0.034	0.68	0.003	Up
AT1G52060	Mannose-binding lectin superfamily protein	−0.59	0.029	−0.45	0.020	Down
AT1G54890	Late embryogenesis abundant protein-like protein	−0.53	0.032	−0.53	0.005	Down
AT2G25980	Mannose-binding lectin superfamily protein	−0.45	0.048	−0.37	0.047	Down
AT3G46230	HSP17.4, Heat shock protein	−5.87	0.034	−1.20	0.032	Down

a Gene descriptions are according to The National Center for Biotechnology Information (https://www.ncbi.nlm.nih.gov/).

Abbreviations: N/A, non-available.

For Gene Ontology (GO) enrichment analysis, the GO slims annotated at the Arabidopsis Information Resource (TAIR) (https://www.arabidopsis.org/portals/genAnnotation/functional_annotation/go.jsp), which are high-level terms from each GO hierarchy that are useful for grouping genes into broad categories (Berardini et al. 2004), were used. The ratio of annotated GO slims in categories of biological process and molecular function, were compared to those in the whole genome (**Tables 3**, **4**, **5**, **6**). In the enriched GO slims for upregulated DEGs in WT plants (**Table 3**), ‘response to stress’ and ‘response to chemical’ include 3 and 4 DEGs, respectively. In the enriched GO slims for downregulated DEGs in WT plants (**Table 4**), ‘response to stress’, ‘response to chemical’ and ‘response to external stimulus’ include 8, 5 and 8 DEGs, respectively. These results indicated that downregulated DEGs are more enriched in GOs related to stress or stimulus responses compared to the upregulated DEGs in WT plants. While in the enriched GO slims for upregulated DEGs in *lym3* (**Table 5**), ‘response to chemical’, ‘response to stress’, ‘response to external stimulus’ and ‘response to biotic stimulus’ include 6, 8, 5 and 5 DEGs, respectively. In the enriched GO slims for downregulated DEGs in *lym3* (**Table 6**), ‘response to chemical’, ‘response to stress’ and ‘response to abiotic stimulus’ include 1, 2 and 2 DEGs, respectively. These results indicated that upregulated DEGs are more enriched in GOs related to the responses to stress or stimulus responses compared to the downregulated DEGs in *lym3* plants.

**Table 3:** Significantly enriched GO slims of upregulated DEGs in WT plants by *B. pumilus* TUAT1 inoculation (*χ*^2^ test, *P* ≤ 0.05)

	Total gene count				Gene count			
Category	Whole genome	DEGs in WT	GO slim	GO ID	Whole genome	DEGs in WT	Corresponding DEGs	*P*-value
Molecular function	45,980	21	Hydrolase activity	GO:0016787	2,701	3	AT4G10540	0.00149
							AT4G20070	
							AT5G40010	
			DNA binding	GO:0003677	1,858	2	AT1G69490	0.00921
							AT4G38340	
			Nucleic acid binding	GO:0003676	1,243	2	AT1G69490	0.01342
							AT4G38340	
			DNA binding transcription factor activity	GO:0003700	1,649	2	AT1G69490	0.01046
							AT4G38340	
			Other binding	GO:0005488	3,417	2	AT1G2910	0.00360
							AT5G38910	
Biological process	124,030	55	Response to stress	GO:0006950	6,044	4	AT1G21520	0.01373
							AT1G62280	
							AT3G46230	
							AT5G40010	
			Anatomical structure development	GO:0048856	5,777	3	AT1G24881	0.02454
							AT1G69490	
							AT5G40010	
			Multicellular organism development	GO:0007275	5,110	3	AT1G24881	0.02867
							AT1G69490	
							AT5G40010	
			Response to chemical	GO:0042221	5,580	3	AT1G62280	0.02569
							AT3G46230	
							AT5G40010	

**Table 4:** Significantly enriched GO slims of downregulated DEGs in WT plants by *B. pumilus* TUAT1 inoculation (*χ*^2^ test, *P* ≤ 0.05)

	Total gene count				Gene count			
Category	Whole genome	DEGs in WT	GO slim	GO ID	Whole genome	DEGs in WT	Corresponding DEGs	*P*-value
Molecular function	45,980	23	Hydrolase activity	GO:0016787	2,701	4	AT1G17710	0.00065
							AT1G73010	
							AT3G10720	
							AT2G43610	
			DNA binding	GO:0003677	1,858	1	AT3G25790	0.04056
			DNA binding transcription factor activity	GO:0003700	1,649	1	AT3G25790	0.04640
			Other binding	GO:0005488	3,417	1	AT1G68740	0.01523
Biological process	124,030	81	Response to stress	GO:0006950	6,044	8	AT1G68740	0.00661
							AT1G73010	
							AT3G09922	
							AT3G10720	
							AT3G25790	
							AT5G09980	
							AT5G20150	
							AT5G20790	
			Anatomical structure development	GO:0048856	5,777	4	AT2G43610	0.02849
							AT3G10720	
							AT4G25160	
							AT5G14330	
			Multicellular organism development	GO:0007275	5,110	4	AT2G43610	0.03332
							AT3G10720	
							AT4G25160	
							AT5G14330	
			Response to chemical	GO:0042221	5,580	5	AT1G73010	0.02089
							AT3G10720	
							AT5G09980	
							AT5G20150	
							AT5G20790	
			Response to external stimulus	GO:0009605	3,255	8	AT1G68740	0.01242
							AT1G73010	
							AT3G09922	
							AT3G10720	
							AT3G25790	
							AT5G09980	
							AT5G20150	
							AT5G20790	

**Table 5:** Significantly enriched GO slims of upregulated DEGs in *lym3* by *B. pumilus* TUAT1 inoculation (*χ*^2^ test, *P* ≤ 0.05)

	Total gene count				Gene count (number)			
Category	Whole genome	DEGs in *lym3*	GO slim	GO ID	Whole genome	DEGs in *lym3*	Corresponding DEGs	*P*-value
Molecular function	45,980	26	Other binding	GO:0005488	3,417	4	AT2G01520	0.00073
							AT2G01530	
							AT4G20260	
							AT1G72150	
			Protein binding	GO:0005515	5,729	4	AT1G48490	0.00019
							AT1G72150	
							AT2G23030	
							AT4G20260	
			Catalytic activity	GO:0003824	5,152	3	AT2G23030	0.00075
							AT1G05010	
							AT1G48490	
			Transferase activity	GO:0016740	3,365	3	AT2G23030	0.00214
							AT1G48490	
							AT1G74590	
			Kinase activity	GO:0016301	1,284	2	AT2G23030	0.02204
							AT1G48490	
			Hydrolase activity	GO:0016787	2,701	2	AT3G13750	0.00920
							AT4G34920	
Biological process	124,030	80	Response to chemical	GO:0042221	5,580	6	AT2G01520	0.01431
							AT4G20260	
							AT4G34920	
							AT1G07620	
							AT1G05010	
							AT1G74590	
			Response to stress	GO:0006950	6,044	8	AT2G01520	0.00639
							AT2G01530	
							AT2G23030	
							AT4G20260	
							AT4G34920	
							AT1G07620	
							AT1G48490	
							AT1G70850	
			Response to external stimulus	GO:0009605	3,255	5	AT3G01290	0.03516
							AT4G20260	
							AT1G07620	
							AT1G05010	
							AT1G48490	
			Response to biotic stimulus	GO:0009607	2,840	5	AT3G01290	0.03879
							AT4G20260	
							AT1G07620	
							AT1G05010	
							AT1G48490	

**Table 6:** Significantly enriched GO slims of downregulated DEGs in *lym3* by *B. pumilus* TUAT1 inoculation (*χ*^2^ test, *P* ≤ 0.05)

	Total gene count				Gene count (number)			
Category	Whole genome	DEGs in *lym3*	GO slim	GO ID	Whole genome	DEGs in *lym3*	Corresponding DEGs	*P*-value
Molecular function	45,980	4	Protein binding	GO:0005515	5,729	1	AT3G46230	0.00002
Biological process	124,030	8	Response to chemical	GO:0042221	5,580	1	AT3G46230	0.00355
			Response to stress	GO:0006950	6,044	2	AT1G52060	0.00011
							AT3G46230	
			Response to abiotic stimulus	GO:0009628	3,412	2	AT1G52060	0.00020
							AT3G46230	

### Confirmation of *LYM3* transcript in *lym3-1* and *lym3-2* mutants

Willmann et al. (2011) demonstrated no *LYM3* transcript in both *lym3-1* and *lym3-2* plants by RT-PCR using primers amplifying the entire coding region of *LYM3* gene. As *lym3-1* and *lym3-2* did not act similarly in RNA-seq analysis, we assumed the lack of *LYM3* transcripts in either *lym3-1* or *lym3-2* plants. We confirmed this by RT-PCR using the same primers as in Willmann et al. (2011) with different PCR cycles such as 25, 30 and 35 cycles. With 25 and 30 cycles, LYM3 transcripts were not amplified even in WT plants. With 35 cycles, WT plants showed clear bands, while *lym3-1* mutant showed faint bands (**Fig. 3C**). There was no amplification in *lym3-2* mutant. The quantification of the band intensities showed that lower amounts of *LYM3* transcripts exist in *lym3-1* mutant compared to WT, while no transcript of *LYM3* was detected in *lym3-2*. As T-DNA is inserted in the first intron of *lym3-1* while it is in the exon in *lym3-2* (Willmann et al. 2011), it is likely that *LYM3* gene was transcribed in *lym3-1* at lower levels compared to WT, although this was not detected by Willmann et al. (2011) with their RT-PCR condition. Variations of RNA-seq results in *lym3-1* and *lym3-2* may be because of the difference in the *LYM3* transcript. Even though *LYM 3* transcript remained at lower amounts in *lym3-1*, Willmann et al. (2011) observed the same phenotype as defects in perception of pathogen PGNs in both *lym3-1* and *lym3-2*. Therefore, we decided to select common DEGs in both alleles for genes related to perceive PGNs of *B. pumilus* TUAT1.

### Response of flg-22 in the presence of *B. pumilus* TUAT1 spores or PGNs on *lym3* mutant plants

**Fig. 4:**
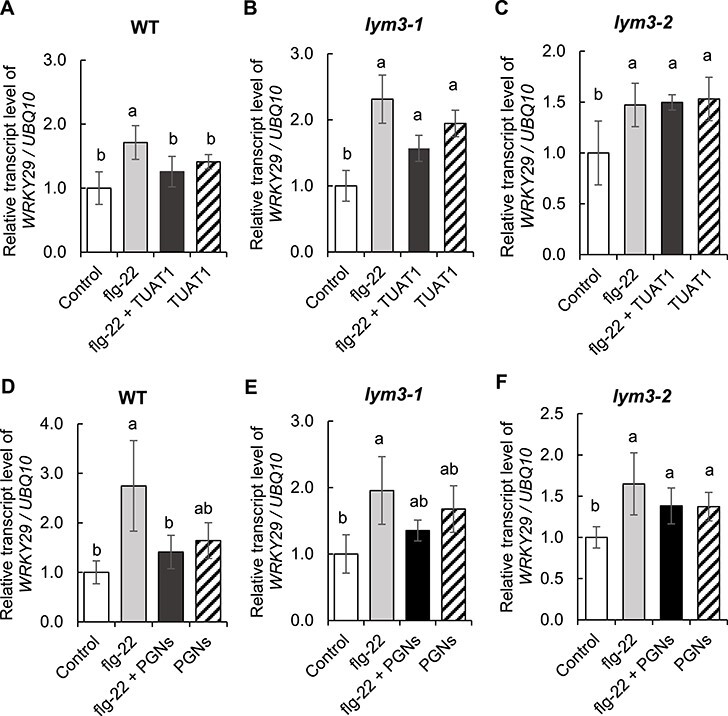
Effects of *B. pumilus* TUAT1 variable spores and PGNs on *WRKY 29* gene expression by flg-22. Plants were treated with flg-22 with or without *B. pumilus* TUAT1 spores as well as PGNs for 1 h. The control plants were treated with neither *B. pumilus* TUAT 1, flg-22 nor PGNs. Relative expression of *WRKY29* normalized to *UBQ10* is shown as the value of the control in each genotype as 1. WT (A), *lym3-1* (B) and *lym3-2* (C) plants were treated with flg-22 with or without TUAT1 spores, and WT (D), *lym3-1* (E) and *lym3-2* (F) plants were treated with flg-22 with or without PGNs. Data are shown as the mean ± SD from three independent biological replicates. Different letters indicate a significant difference by Tukey’s test (*P* ≤ 0.05).

Results of RNA-seq analysis suggested a possibility that *B. pumilus* TUAT inoculation alleviated defense responses in WT plants but enhanced them in *lym3* mutant plants. To confirm this, changes in mRNA abundance of the defense-responsive gene *WRKY29* (Liang et al. 2013) were analyzed in both WT and *lym3* mutant plants after treatment with an elicitor, flg-22, with or without *B. pumilus* TUAT1 viable spore pretreatment as well as purified PGNs. The relative transcriptional level of *WRKY29* was significantly increased by flg-22 treatment in both WT and *lym3* mutant plants (**Fig. 4A**–**F**). In WT plants, *WRKY29* expression was unchanged compared to the control by the simultaneous application of *B. pumilus* TUAT1 spores and flg-22 (**Fig. 4A**), while in *lym3-1* and *lym3-2* mutant plants, *WRKY29* expression was increased by the simultaneous application of *B. pumilus* TUAT1 spores and flg-22 compared to the control to almost the same level of single flg-22 application (**Fig. 4B, C**). By the single application of *B. pumilus* TUAT1 spores, expression of *WRKY29* gene was not changed compared to the control in WT plants (**Fig. 4A**), while it was increased in *lym3* mutant plants (**Fig. 4B, C**). The similar trends were also observed when purified PGNs from *B. pumilus* TUAT1 were used in place of the spores. In WT plants, *WRKY29* expression was unchanged compared to the control by the simultaneous application of *B. pumilus* TUAT1 PGNs and flg-22 (**Fig. 4D**), while in *lym3-1* and *lym3-2* mutant plants, *WRKY29* expression was increased by the simultaneous application of *B. pumilus* TUAT1 PGNs and flg-22 to almost the same level of single flg-22 application (**Fig. 4E, F**).

## Discussion

### Involvement of PGN receptor for growth promotion in *Arabidopsis* treated with TUAT1 spores and PGNs

Growth promotion by inoculating *B. pumillus* TUAT1 has been observed in various plants (Win et al. 2018, 2019, 2020, Agake et al. 2021, 2022a, 2022b, Hasibuan et al. 2021, Borjigin et al. 2024), and spores are more effective than vegetative cells (Ngo et al. 2019). *Bacillus* spores carry a thick PGN layer in their cell wall, so we hypothesized that perception of *B. pumillus* TUAT1 PGNs by plant receptors is involved in the mechanism of growth promotion by this strain. Consistent with our hypothesis, purified PGNs, as well as spores of *B. pumillus* TUAT1, promoted growth of WT *Arabidopsis thaliana* (**Fig. 2**). PRRs work as a master switch to initiate the signaling pathway by recognizing MAMPs and pathogen-associated molecular patterns like PGNs and flagellin and then transmitted the signal into the inside of the cell with the help of RLKs (Zhang et al. 2019). In the present study, we investigated whether PGN receptors for pathogens, i.e. LYM1, LYM3 and CERK1 (Willmann et al. 2011), are involved in perception of *B. pumillus* TUAT1 using *A. thaliana* mutants of *lym1, lym3* and *cerk1*.

In the first study, root growth promotion by *B. pumillus* TUAT1 spores was observed in *lym1-1, lym1-2* and *cerk1-2*, but not in *lym3-1* and *lym3-2* mutant plants, suggesting that LYM3 protein is required for the recognition of *B. pumilus* TUAT1 spores to promote growth (**Fig. 1**). Similarly, growth promotion by purified PGNs of *B. pumilus* TUAT1 was observed in WT, but not in *lym3* mutant plants (**Fig. 2**), suggesting that LYM3 receptor protein is required for growth promotion by recognizing PGNs of *B. pumilus* TUAT1 strain. Since the LYM3 receptor protein lacks a cytoplasmic kinase domain, additional proteins are likely required for transmitting the signal inside of the cell after the perception of *B. pumilus* TUAT1. Regarding the perception of pathogen PGNs, signaling perceived by LYM3 with LYM1 is transmitted to CERK1. PGP effects by *B. pumillus* TUAT1 spores were observed in both allelic *lym1* mutant plants, indicating that LYM1 is not involved in the response to this strain. As for CERK1, PGP effects were observed in *cerk1-2*. We also tried to test another allelic mutant, *cerk1-1* with Nossen background (Miya et al. 2007) but could not evaluate the result because WT of Nossen did not respond to *B. pumillus* TUAT1 (data not shown). Thus, possibility of involvement of CERK1 on PGP effects remains and has to be confirmed in further study. Whether LYM1 and CERK1 are involved together with LYM3 may be the critical point to discriminate responses by PGPR from those by pathogens.

### The enriched slim GOs of genes regulated by *B. pumilus* TUAT1 inoculation

In DEGs of the *lym3* mutant, all enriched GO slims of the biological process category were related to response to stress and stimulus, such as ‘response to chemical’, ‘response to stress’, ‘response to external stimulus’ and ‘response to biotic stimulus’, and upregulated DEGs are more enriched than downregulated DEGs in these GO slims (**Tables 5**, **6**). In contrast, in WT plants, downregulated DEGs are more enriched than upregulated DEGs in GO slims related to stress or stimulus responses, such as ‘response to external stimulus’, ‘response to stress’ and ‘response to chemical’ (**Tables 3**, **4**). This means that upregulated DEGs are more enriched in *lym3*, while downregulated DEGs are more enriched in WT plants in GO slims related to stress or stimulus responses. The numbers of DEGs affected by *B. pumilus* TUAT1 inoculation were much higher in *lym3-1* and *lym3-2* compared to WT plants (**Fig. 3**, **Supplementary Fig. S1**), which may be because stress responses due to the decrease or lack of the *LYM3* transcripts in *lym3* mutants (**Fig. 3C**) affected expression of more genes compared in WT plants.

To pay attention to the broader overview of transcriptional reprogramming, we also performed GO enrichment analysis for genes in cluster 2 and cluster 7 of the heatmap (**Supplementary Table S4** and **S5**) as they showed similar expression patterns in the two allelic *lym3-1* and *lym3-2* mutant plants and the patterns are opposite to those in WT plants. In cluster 2 where genes were upregulated by inoculation of *B. pumilus* TUAT1 in WT and downregulated in *lym3-1* and *lym3-2*, no stress-related GO slims were enriched (**Supplementary Table S4**). In cluster 7 where genes were downregulated by inoculation of *B. pumilus* TUAT1 in WT and upregulated in *lym3-1* and *lym3-2*, the GO slim of ‘response to chemical’ was enriched (**Supplementary Table S5**). This also supports that stress response is alleviated in WT and induced in *lym3* mutant plants by *B. pumilus* TUAT1 spores.

### Involvement of LYM3 in alleviation of defense response by *B. pumilus* TUAT1 and PGNs

The results of RNA-seq suggest that *B. pumilus* TUAT1 spores act as a regulator to reduce defense-related gene expression and that LYM3 is required for this regulation. Supporting this hypothesis, *B. pumilus* TUAT1 alleviated the induction of the defense-responsive gene *WRKY29* by flg-22 treatment in WT plants, but not in *lym3-1* and *lym3-2* mutant plants (**Fig. 4A–C**). The expression of *WRKY29* gene was also significantly reduced by flg-22 treatment in the presence of PGNs in WT plants, but this trend was not observed in *lym3-1* and *lym3-2* mutant plants (**Fig. 4D–F**). These similar results of flg-22 treatment in the presence of PGNs and *B. pumilus* TUAT1 spores indicate that LYM3 receptor protein is required for recognizing PGNs of *B. pumilus* TUAT1 spores to alleviate defense responses.

Previously, Liang et al. (2013) demonstrated that rhizobium Nod factors, the signaling molecules for legume infection structurally similar chitotetraose, have the ability to suppress plant innate immunity in both legumes and non-legumes. The same paper showed that flg-22 triggered ROS production and induction of the *WRKY29* gene and other defense responses were significantly reduced by pretreatment with Nod factors in both soybean and *Arabidopsis*. LYK3, which is a LysM-containing receptor kinase, is required for Nod factor suppression of flg-22-triggered immunity. It is possible that rhizobium Nod factors and PGNs in *B. pumilus* TUAT1 have similar roles in the suppression of defense responses through LysM-containing receptors or receptor kinases. Indeed, similar to our findings with PGN, it was also shown that treatment of plants with Nod factor stimulated root growth in maize and other plants (e.g. Tanaka et al. 2015, Buendia et al. 2019).

It is known that plant growth is negatively correlated with the induction of defense responses, and interactions between plant hormones and MAMP-triggered immunity are suggested in the mechanisms of this trade-off (Lozano-Durán et al. 2013, Huot et al. 2014). The present results are consistent with a model where PGNs of *B. pumilus* TUAT1 are recognized by LYM3 receptor protein, alleviating defense responses, which results in growth promotion.

## Materials and Methods

### Screening of *Arabidopsis* T-DNA knockout mutants for *LYM1*, *LYM3* and *CERK1*

*Arabidopsis thaliana* plants were grown on rockwool in a plastic container at 22°C under a 16-h light (150 μmol m^−2^ s^−1^)/8-h dark photoperiod using Molecular Genetics Research Laboratory hydroponic solution as a nutrient source (Fujiwara et al. 1992, Hirai et al. 1995).

*lym3-1* (stock number SALK_111212C) and *lym3-2* (SALK_132566C) provided by the Salk Institute (Alonso et al. 2003), *lym1-1* (GK419G07) and *cerk1-2* (GK-096F09) provided by GABI-kat (https://www.gabi-kat.de/) and *lym1-2* (SK17791) (Robinson et al. 2009) are ecotype Columbia-0 and obtained from the Arabidopsis Biological Resource Center (https://abrc.osu.edu/stocks/). The *lym3-1* and *lym3-2* mutants with the insertion of a T-DNA in the first exon of *LYM3* (AT1g77630), *lym1-1* and *lym1-2* mutants with the insertion of a T-DNA in the second and fifth exon of the *LYM1* gene (AT1G21880), respectively, and *cerk1-2* with the insertion of a T-DNA in the intron of the *CERK1* gene (At3g21630) were reported by Willmann et al. (2011).

Gene-specific primers and T-DNA left border primers for screening mutant plants carrying each T-DNA in homozygous states are listed in **Supplementary Table S2**. The T-DNA left border primer pROKr3 (Lin and Oliver 2008) was used for *lym3-1* and *lym3-2*, GABI-8409 (https://www.gabi-kat.de/) for *lym1-1* and *cerk1-2* and pSK-3 (Robinson et al. 2009) for *lym1-2*.

### Measurement of growth promotion effects by *B. pumilus* TUAT1 and PGNs on selected mutant lines

*Bacillus pumilus* TUAT1 cultures were grown as previously described (Agake et al. 2022b). Vegetative cells of *B. pumilus* TUAT1 were cultured in 300 ml of trypticase soy (TSB) broth in one litter Erlenmeyer flask at 180 rpm, 30°C for 24 h. A high concentration of spores was obtained by culturing for 72 h in Difco sporulation medium instead of TSB (Nicholson and Setlow 1990). The cells were collected by centrifugation, washed with sterilized water purified with reverse osmosis (RO) membrane several times and finally resuspended in sterilized RO water. Spore preparations were incubated at 65°C for 1 h to kill vegetative cells. The colony forming units (CFU) of the inoculant cultures were confirmed by plating onto T-soy agar.

Plant culture and inoculation with the *B. pumilus* TUAT1 strain were performed as follows. Seeds were surface-sterilized by immersion in 70% ethanol for 10 min and 1% NaClO for 10 min, washed several times with sterilized water and then sown on germination medium (Umezawa et al. 2009). The seeds were vernalized at 4°C in the dark for 2–3 d and then transferred into a growth chamber with a photoperiod of 16-h light (150 μmol m^−2^ s^−1^ photon flux density) and 8-h darkness at 22°C. Two weeks after germination, seedlings were transplanted into plastic trays (15 wells/tray) containing 72 g of autoclaved soil (Shinano Baiyoudo Co., Ltd., Nagano, Japan) per well and then grown in the growth chamber set as described earlier. The Shinano soil contains approximately 375 mg N kg^−1^, 750 mg P_2_O_5_ kg^−1^ and 375 mg K_2_O kg^−1^. The plants were fertilized initially using 625 ml of liquid fertilizer diluted 2000 times with sterile RO water in each tray (15 plants) (Hanakoujyo, N:P:K = 8:10:5 mg ml^−1^, Sumitomo Chemical Garden Products Inc., Tokyo, Japan), and subsequently irrigated, as needed, with sterilized RO water. After transplantation, the trays were covered with plastic films to maintain moisture for 1 d. After that, plastic films were removed, and 4 ml of spore suspension of *B. pumilus* TUAT1 at a concentration of 10^7^ CFU ml^−1^ was applied to the soil in each well, where control plants were treated with 4 ml of sterilized RO water.

In case of PGN treatments, PGNs were extracted and purified from *B. pumilus* TUAT1 according to a previous report (Kühner et al. 2014). One milliliter of purified PGNs at a concentration of 300 μg ml^−1^ was applied to a plant grown in soil in each well, where control plants were treated with 1 ml of sterilized RO water. Plants were sampled at 14 d after spore inoculation as well as PGN treatments.

Fresh weight and dry weight of shoots and roots were determined in 15 replicates per treatment. To obtain dry weights, the plant materials were kept in a dryer at 80°C for 48 h. To measure the total root length, lateral root length and root surface area, plant roots were scanned using an Epson Perfection V700 Photo (Seiko Epson Corporation, Nagano, Japan) and analyzed using WinRHIZO software ver. 2004 (Regent Instruments Inc., Quebec, Canada) as described by Haidari et al. (2017).

### RNA-seq and data analysis

Plants were cultured and transplanted onto Shinano soil in plastic trays as described earlier. Three weeks after transplanting, each plant was treated with 4 ml of spore suspension of *B. pumilus* TUAT1 at 10^7^ CFU ml^−1^. The roots were sampled 1 h after inoculation. Roots from five plants were combined to generate one sample, and three biological replicates were prepared from 15 plants. Total RNAs were extracted using an RNeasy plant mini Kit (Qiagen, Hilden, Germany) according to the manufacturer’s instructions. As previously reported (Ju et al. 2017), six libraries were constructed and sequenced by the Beijing Genomics Institute (www.genomics.org.cn BGI, Shenzhen, China). Clean tags were mapped to the reference genome and genes that were available in the Arabidopsis reference genome GCF_000001735.4_TAIR10.1. For gene expression analysis, the matched reads were calculated and then normalized to reads per kilobase of transcript per million mapped reads using the RESM software. The significance of differential gene expression was confirmed with the BGI bioinformatics service using a *Q* value of ≤0.05.

GOs were based on the GO annotations at the Arabidopsis Information Resource (TAIR) (https://www.arabidopsis.org/portals/genAnnotation/functional_annotation/go.jsp) (Berardini et al. 2004). The ratios of annotated GO slims, which are high-level terms from each GO hierarchy that are useful for grouping genes into broad categories (Berardini et al. 2004), in categories of biological process and molecular function, were compared to those in the whole genome.

Identification of genes with differential expression in WT and *lym3* mutants, analysis of volcano plots, PCA, t-SNE plot, dendrogram and *K*-means clustering were performed using online based software iDEP1.1 (Ge et al. 2018). For the identification of DEGs with up- and downregulation, DEGs were defined as the log_2_ fold change (FC) ≥1 and false discovery rate (FDR) was adjusted to ≤0.05.

### Quantification of LYM3 transcripts in *lym3-1* and *lym3-2* mutant plants

The same amount of RNA extracted from roots of 3-weeks-old plants grown on Shinano soil were reverse transcribed using the Prime Script™ RT Reagent Kit with gDNA Eraser (Perfect Real Time) (Takara Bio, Shiga, Japan). cDNA was amplified by GoTaq® DNA Polymerase (Promega, Madison, WI, USA) using primers Lym3-f and Lym3-r (Willmann et al. 2011) or UBQ10_F and UBQ10_R (**Supplementary Table S3**) at 25, 30 and 35 cycles. *LYM3* and *UBQ10* transcript detected at 35 and 30 cycles, respectively, were quantified using GelAnalyzer 23.1 (available at www.gelanalyzer.com) by Istvan Lazar Jr., PhD, and Istvan Lazar Sr., PhD, CSc, and the intensities of LYM3 were normalized by those of *UBQ10*.

### Effect of TUAT1 and PGNs on the induction of *WRKY29* expression by flg-22

The synthesized flg-22 peptide (containing the conserved 22 amino acid sequence of *Pseudomonas aeruginosa*) was purchased from Alpha Diagnostic Intl Inc. (San Antonio, TX, USA). Plants were cultured and transplanted on Shinano soil in plastic trays as described earlier. Three weeks after transplanting, each plant was treated with 4 ml of *B. pumilus* TUAT1 spores suspension at 10^7^ CFU ml^−1^ or sterilized RO water. In the case of purified PGN treatments, each plant was treated with 1 ml of PGNs at a concentration of 300 µg ml^−1^ or sterilized RO water. Immediately after treatment with *B. pumilus* TUAT1 spores and PGNs, the plants were treated with 4 ml of 1 µM flg-22. One hour after treatment with flg-22, the roots were sampled, immediately frozen in liquid N_2_ and stored at −80°C until RNA extraction. Sterilized RO water was used as a control treatment instead of *B. pumilus* TUAT1 spores as well as PGNs and flg-22.

Total RNAs were extracted from the roots using a RNeasy plant mini Kit (Qiagen, Hilden, Germany), according to the manufacturer’s instructions. Reverse transcription was performed using the Prime Script™ RT Reagent Kit with gDNA Eraser (Perfect Real Time) (Takara Bio, Shiga, Japan). Quantitative PCR was performed using the KAPA SYBR FAST qPCR Master Mix (2×) Kit (KAPA Biosystems, Wilmington, MA, USA) and Light Cycler 96 (Roche Diagnostics, Basel, Switzerland) following the manufacturer’s protocols. The *WRKY29* and *UBQ10* genes were used as the target gene and internal control, respectively, using the same primers used by Liang et al. (2013) (**Supplementary Table S3**). Relative expression levels were calculated relative to the calculated values using the 2^-ΔΔC^T method (Livak and Schmittgen 2001).

## Supplementary Material

pcae016_Supp

## Data Availability

The generated mutants analyzed in this article will be shared on request to the corresponding author. RNA-seq data have been deposited with links to BioProject accession number PRJDB17142 in the DDBJ BioProject database.
