# Supplementary material for: Involvement of Peptidoglycan Receptor Proteins in Mediating the Growth-Promoting Effects of Bacillus pumilus TUAT1 in Arabidopsis thaliana
Source: Plant Cell Physiol. 2024 Feb 19;65(5):748–61. doi: 10.1093/pcp/pcae016 (PMC11138354; doi:10.1093/pcp/pcae016)
Supplement: pcae016_Supp [file pcae016_supp.zip › suppl_data/pcp-2023-e-00290-File007.pdf]

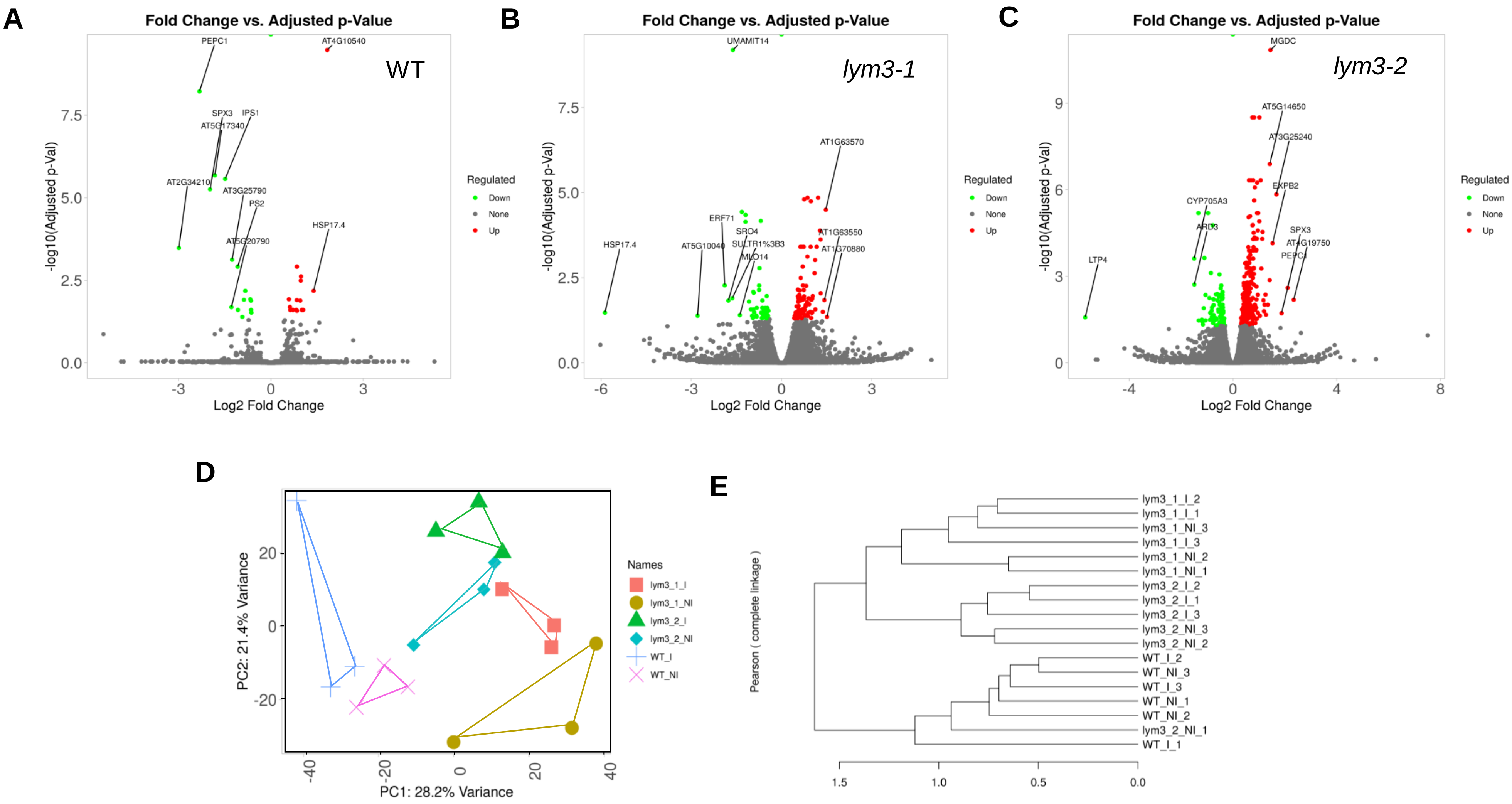

Supplementary Fig S1. The volcano plots showing distribution of DEGs treated with *B. pumilus* TUAT1 in WT (A), *lym3-1* (B) and *lym3-2* (C). The log2 fold change is plotted on the x-axis, and the negative log10 (FDR) (p-value) is plotted on the y-axis. The green, red and gray points show downregulated DEGs, upregulated DEGs and non-regulated genes, respectively. (D) PCA represent the multivariate of DEGs by *B. pumilus* TUAT1 inoculation. . (E) A dendrogram among samples using genes with maximum expression level at the top 75%.

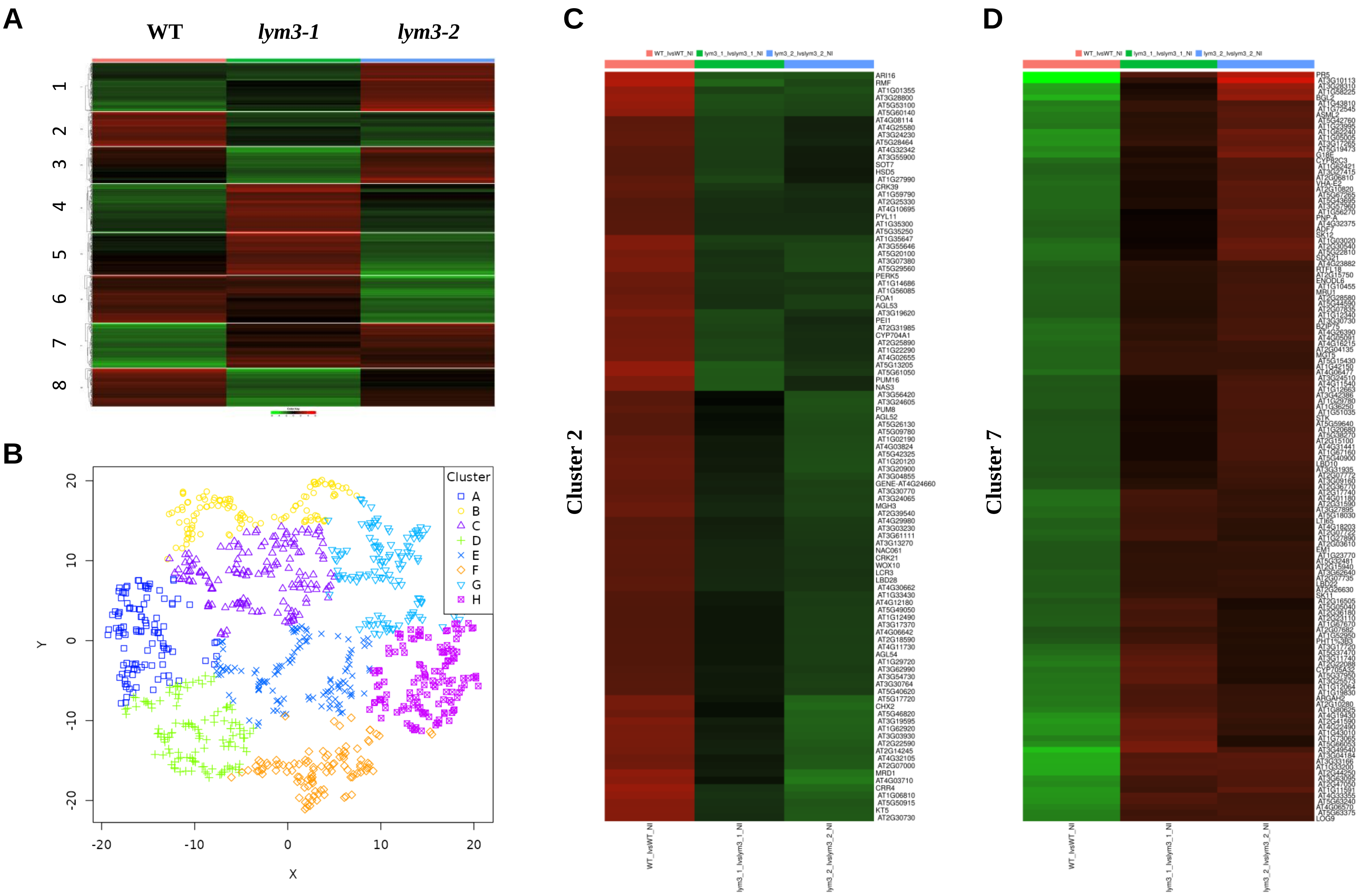

Supplementary Fig S2. (A) Heatmap showing the expression levels of the top 1000 genes demonstrating increased or decreased trends and (B) t-SNE plots demonstrating dimension reduction and highlighting the presence of 8 clusters. (C) and (D) are zoomed-in sections of heatmap into cluster 2 and 7, respectively.

Supplementary table S1. Summary of the sequence assembly after RNA-seq.

Sample	Row reads number ($x10^6$)	Row data size ($x10^9$) bp	Clean reads number ($x10^6$)	Clean data size ($x10^9$) bp	Total Mapped Reads (%)	Unique Match(%)
WT_ I	48.10	9.62	44.68	8.94	94.90	93.50
WT _NI	48.93	9.79	45.38	9.08	94.83	93.45
lym3-1 _I	42.32	8.46	39.07	7.81	94.67	92.97
lym3-1 _NI	30.48	6.10	27.98	5.60	94.79	92.87
lym3-2 _I	45.48	9.10	42.86	8.57	96.08	94.59
lym3-2 _NI	43.91	8.78	40.53	8.11	96.08	94.57

Supplementary table S2. Gene specific primers and T-DNA left border primers used for screening *lym1*, *lym3* and *cerk1* mutants.

Primers	Sequence (5`to 3`)	References
AT1g77630_F	CTTAGCATCAATGGCGAC	This study
AT1g77630_R	CTTACCTAGCAATGGGACAGC	This study
AT1G21880_F	CTCTCAGCTTGTGCTTCAAAG	This study
AT1G21880_R	GTAGCTAACTGAGGAGGCAGAG	This study
At3g21630_F	GACAGAAGTTGGAGGTTCAGC	This study
At3g21630_R	CTTTGACGTGAGATGCTGATATC	This study
pROKr3	CCTTTCGCTTTCTTCCCTTCCTTTCT	Lin and Oliver, 2008
pSKTAILL3	ATACGACGGATCGTAATTTGTCG	Robinson et al, 2003
GABI-O8409	ATATTGACCATCATACTCATTGC	https://www.gabi-kat.de/

Supplementary table S3. Primers used for RT-PCR and quantitative RT-PCR.

Primers	Sequence (5`to 3`)	References
WRKY29_F	ATCCAACGGATCAAGAGCTG	Liang et al. (2013)
WRKY29_R	GCGTCCGACAACAGATTCTC	Liang et al. (2013)
UBQ10_F	GGCCTTGTATAATCCCTGATGAATAAG	Liang et al. (2013)
UBQ10_R	AAAGAGATAACAGGAACGGAAACATAGT	Liang et al. (2013)
Lym3-f	ATGAAGAATCCAGAAAAACC	Williams et al. (2011)
Lym3-r	TTAGAAAACAAAAAAGCAAG	Williams et al. (2011)

Supplementary table S4. Significantly enriched GO slims of genes in the cluster 2 of the heatmap (χ^2 test, $P \leq 0.05$).

Category	Total gene count		GO Slim	GO ID	Gene count (no.)		Corresponding genes	P-value
	Whole genome	Cluster 2			Whole genome	Cluster 2		
Molecular function	45980	72	Other binding	GO:0005488	3417	3	AT1G02190<br>AT1G14686<br>AT5G29560	0.01603
			Protein binding	GO:0005515	5729	7	AT1G12490<br>AT1G14686<br>AT1G22290<br>AT1G59790<br>AT2G31985<br>AT5G50915<br>AT5G60140	0.00087
			Catalytic activity	GO:0003824	5152	7	AT1G01355<br>AT1G02190<br>AT1G29720<br>AT2G30730<br>AT3G24230<br>AT3G30770<br>AT5G29560	0.00123
			Transferase activity	GO:0016740	3365	4	AT1G29720<br>AT1G33430<br>AT2G22590<br>AT2G30730	0.01117
			RNA binding	GO:0003723	2519	2	AT3G17370<br>AT3G61111	0.04218
			Hydrolase Activity	GO:0016787	2701	3	AT1G20120<br>AT3G19620<br>AT4G11730	0.02505
Biological process	124030	100	Biosynthesis process	GO:0009058	7969	4	AT1G02190<br>AT1G33430<br>AT5G42325<br>AT5G50915	0.02243
			Nucleobase-containing compound metabolic process	GO:0006139	6050	3	AT3G30770<br>AT5G42325<br>AT5G50915	0.00639

Supplementary table S5. Significantly enriched GO slims of genes in the cluster 7 of the heatmap (χ^2 test, $P \leq 0.05$).

Category	Total gene count		GO Slim	GO ID	Gene count (no.)		Corresponding gens	P-value
	Whole genome	Cluster 7			Whole genome	Cluster 7		
Molecular function	45980	87	Other binding	GO:0005488	3417	4	AT2G36180<br>AT3G17720<br>AT4G23882<br>AT4G26390	0.01419
			Protein binding	GO:0005515	5729	6	AT1G03020<br>AT1G23770<br>AT2G17740<br>AT2G30540<br>AT4G01180<br>AT5G15430	0.00187
			Catalytic activity	GO:0003824	5152	3	AT1G27890<br>AT1G29780<br>AT3G17720	0.00679
			Transferase activity	GO:0016740	3365	3	AT2G36770<br>AT4G26390<br>AT5G37950	0.02033
			Hydrolase Activity	GO:0016787	2701	3	AT1G29780<br>AT4G32375<br>AT5G22810	0.03085
Biological process	124030	112	Response to chemical	GO:0042221	5580	6	AT1G03020<br>AT1G19830<br>AT1G67160<br>AT2G17740<br>AT2G47050<br>AT5G18030	0.02642
			Biosynthesis process	GO:0009058	7969	3	AT1G23995<br>AT1G52950<br>AT3G10113	0.03288
			Nucleobase-containing compound metabolic process	GO:0006139	6050	7	AT1G23995<br>AT1G27890<br>AT1G52950<br>AT3G09160<br>AT3G10113<br>AT4G01180<br>AT4G26390	0.01833

Supplementary table S6 Gene list in the cluster 2 of the heatmap.

Locus Tag	Gene description	Z score in WT	Z score in *lym3-1*	Z score in *lym3-2*
AT3G61730	RMF, Reduced male fertility	3.2668	-1.9214	-1.3454
AT5G08730	ARI16, IBR domain-containing protein	3.1697	-1.6134	-1.5562
AT2G45350	CRR4, Pentatricopeptide repeat (PPR) superfamily protein	2.8443	-0.8711	-1.9732
AT3G28800	Pseudo	2.8636	-1.4987	-1.3649
AT1G53480	MRD1, *mto1* responding down 1	2.6671	-0.4767	-2.1903
AT4G03710	Pseudo	2.5525	-0.2520	-2.3005
AT5G53100	NAD(P)-binding rossmann-fold superfamily protein	2.7651	-1.4531	-1.3120
AT1G06810	Endonuclease/glycosyl hydrolase	2.7232	-0.9416	-1.7816
AT5G61050	Histone deacetylase-related / HD-like protein	2.7327	-1.6797	-1.0529
AT1G01355	Putative endonuclease or glycosyl hydrolase	2.7445	-1.2519	-1.4926
AT5G13205	Pseudo	2.7089	-1.7239	-0.9850
AT5G60140	AP2/B3-like transcriptional factor family protein	2.6516	-1.3903	-1.2613
AT4G37553	KT5, ncRNA	2.5202	-0.8478	-1.6724
AT2G30730	Protein kinase superfamily protein	2.4930	-0.8013	-1.6917
AT5G50915	Basic helix-loop-helix (bHLH) DNA-binding superfamily protein	2.5009	-0.8758	-1.6251
AT3G19595	Haloacid dehalogenase-like hydrolase (HAD) superfamily protein	2.3725	-0.5116	-1.8609
AT1G09240	NAS3, Nicotianamine synthase 3	2.3745	-1.6819	-0.6926
AT1G79400	CHX2, Cation/H^+ exchanger 2	2.2018	-0.1958	-2.0060
AT3G55646	TPRXL, Tetrapeptide repeat homeobox like pseudogene	2.4223	-1.0975	-1.3248
AT5G59280	PUM16, Pumilio 16	2.3491	-1.6645	-0.6846
AT1G35647	Pseudo	2.4056	-1.2028	-1.2028
AT3G03930	Kinase-like protein	2.2582	-0.4688	-1.7894
AT1G62920	Proteasome maturation factor	2.2416	-0.4198	-1.8219
AT5G17720	Alpha/beta-Hydrolases superfamily protein	2.2054	-0.3272	-1.8783
AT2G22590	UDP-Glycosyltransferase superfamily protein	2.2376	-0.5402	-1.6975
AT3G07380	Glycosyltransferase family protein (DUF23)	2.2924	-0.9532	-1.3392
AT5G29560	Caleosin-related family protein	2.2862	-0.9157	-1.3705
AT3G19620	Glycosyl hydrolase family protein	2.2821	-1.3816	-0.9005
AT2G14245	Pseudo	2.1928	-0.5717	-1.6211
AT2G07000	Uncharacterized protein	2.1303	-0.4136	-1.7167
AT5G46820	Carboxyl-terminal proteinase-like protein, putative (DUF239)	2.0310	-0.1958	-1.8353
AT5G20100	plant/protein	2.2014	-0.8920	-1.3094
AT4G32105	Beta-1,3-N-Acetylglucosaminyltransferase family protein	2.0630	-0.3650	-1.6981

AT1G22290	14-3-3 family protein	2.1482	-1.3336	-0.8146
AT4G02655	Uncharacterized protein	2.1191	-1.2685	-0.8507
AT2G25890	Oleosin family protein	2.1065	-1.3002	-0.8063
AT2G31985	lipoprotein (DUF1264)	2.0608	-1.2779	-0.7829
AT5G27070	AGL53, AGAMOUS-like 53	2.0640	-0.8708	-1.1932
AT2G44890	CYP704A1, cytochrome P450, family704, subfamily A, polypeptide1	2.0575	-1.2216	-0.8359
AT5G07500	PEI1, Zinc finger C-x8-C-x5-C-x3-H type family protein	2.0337	-1.3365	-0.6971
AT3G17320	FOA1, F-box and associated interaction domains-containing protein	2.0518	-0.8647	-1.1871
AT1G20120	GDSL-like Lipase/Acylhydrolase superfamily protein	1.9267	-0.4446	-1.4821
AT3G20900	Uncharacterized protein	1.9000	-0.4009	-1.4990
AT1G14686	ENTH/ANTH/VHS superfamily protein	1.9953	-0.9368	-1.0585
AT4G34440	PERK5, Protein kinase superfamily protein	1.9930	-1.0242	-0.9688
AT1G19890	MGH3, male-gamete-specific histone H3	1.9643	-0.6981	-1.2662
AT2G39540	Gibberellin-regulated family protein	1.9514	-0.6985	-1.2529
AT5G42325	Transcription factor IIS protein	1.8590	-0.3890	-1.4700
AT3G04855	Uncharacterized protein	1.8885	-0.5199	-1.3687
AT1G02190	Fatty acid hydroxylase superfamily	1.8337	-0.4021	-1.4316
AT4G03824	Pseudo	1.8228	-0.3781	-1.4447
AT1G56085	Cyclophilin	1.9072	-0.9222	-0.9850
AT1G22240	PUM8, Pumilio 8	1.7484	-0.2321	-1.5163
AT3G24065	Plant self-incompatibility protein S1 family	1.8613	-0.6397	-1.2216
AT4G24660	HB22, Homeobox protein 22	1.8101	-0.5761	-1.2340
AT3G24605	late embryogenesis abundant protein, group 2	1.6323	-0.0750	-1.5574
AT3G30770	Eukaryotic aspartyl protease family protein	1.7858	-0.5164	-1.2694
AT4G04540	CRK39, cysteine-rich RLK (RECEPTOR-like protein kinase) 39	1.8150	-1.0634	-0.7516
AT5G28464	Pseudo	1.7932	-1.1598	-0.6334
AT3G56420	Thioredoxin superfamily protein	1.6302	-0.1201	-1.5101
AT1G27990	Uncharacterized protein	1.7356	-1.3204	-0.4152
AT3G24230	Pectate lyase family protein	1.7751	-1.2012	-0.5739
AT4G25580	CAP160 protein	1.7175	-1.1928	-0.5247
AT4G11250	AGL52, AGAMOUS-like 52	1.5943	-0.1551	-1.4392
AT4G08114	Pseudo	1.6963	-1.1548	-0.5415
AT5G26130	CAP (Cysteine-rich secretory proteins, Antigen 5, and Pathogenesis-related 1 protein) superfamily protein	1.5833	-0.1841	-1.3992
AT5G09780	Transcriptional factor B3 family protein	1.5924	-0.2183	-1.3741

AT1G59790	Cullin family protein	1.7218	-0.9146	-0.8072
AT4G30662	Uncharacterized protein	1.7109	-0.7388	-0.9721
AT3G13270	Pseudo	1.6983	-0.6500	-1.0483
AT3G50510	LBD28, LOB domain-containing protein 28	1.6998	-0.7289	-0.9708
AT3G44350	NAC061, NAC domain containing protein 61	1.6745	-0.6863	-0.9882
AT1G20710	WOX10, WUSCHEL related homeobox 10	1.6729	-0.6753	-0.9977
AT3G30764	Pseudo	1.6065	-0.3814	-1.2251
AT4G23290	CRK21, cysteine-rich RLK (RECEPTOR-like protein kinase) 21	1.6656	-0.6716	-0.9940
AT3G03230	alpha/beta-Hydrolases superfamily protein	1.6461	-0.5547	-1.0914
AT3G54730	Transcription repressor	1.5941	-0.3853	-1.2088
AT4G32342	Uncharacterized protein	1.6025	-1.1525	-0.4500
AT3G61111	Zinc-binding ribosomal protein family protein	1.6264	-0.5663	-1.0601
AT4G29980	Fasciclin-like arabinogalactan protein	1.6134	-0.5093	-1.1041
AT5G40620	Uncharacterized protein	1.5866	-0.4170	-1.1696
AT5G47175	LCR3, low-molecular-weight cysteine-rich 3	1.6344	-0.7027	-0.9317
AT3G55900	F-box family protein	1.5768	-1.1507	-0.4261
AT1G35300	Pseudo	1.6239	-0.8119	-0.8119
AT4G10020	HSD5, Hydroxysteroid dehydrogenase 5	1.5494	-1.1837	-0.3658
AT5G35250	Pseudo	1.6177	-0.8089	-0.8089
AT1G28170	SOT7, sulfotransferase 7	1.5617	-1.1304	-0.4313
AT3G62990	Myelin transcription factor-like protein	1.5555	-0.4425	-1.1130
AT4G11730	Cation transporter/ E1-E2 ATPase family protein	1.5166	-0.3339	-1.1827
AT5G45860	PYL11, PYR1-like 11	1.5934	-0.7967	-0.7967
AT1G29720	Leucine-rich repeat transmembrane protein kinase	1.5081	-0.3296	-1.1784
AT5G27090	AGL54, AGAMOUS-like 54	1.5081	-0.3296	-1.1784
AT1G33430	Galactosyltransferase family protein	1.5189	-0.3743	-1.1446
AT4G06642	Pseudo	1.5009	-0.3260	-1.1749
AT2G18590	Major facilitator superfamily protein	1.4975	-0.3243	-1.1731
AT1G12490	F-box associated ubiquitination effector family protein	1.4901	-0.3206	-1.1695
AT3G17370	Pentatricopeptide repeat (PPR) superfamily protein	1.4901	-0.3206	-1.1695
AT5G49050	Universal stress A-like protein	1.4901	-0.3206	-1.1695
AT4G12180	Pseudo	1.5047	-0.3854	-1.1194
AT4G10695	CDC68-like protein	1.5607	-0.8369	-0.7239
AT2G25330	TRAF-like family protein	1.5557	-0.8343	-0.7214

Gene descriptions are according to the National Center for Biotechnology Information (NCBI, https://www.ncbi.nlm.nih.gov/).

Supplementary table S7 Gene list in the cluster 7 of the heatmap.

Locus Tag	Gene description	Z score in WT	Z score in *lym3-1*	Z score in *lym3-2*
AT3G10113	Homeodomain-like superfamily protein	-4.6602	0.7770	3.8832
AT1G75040	PR5, pathogenesis-related protein 5	-4.8380	1.5441	3.2939
AT3G49540	Uncharacterized protein	-3.5231	2.2614	1.2617
AT3G57260	BGL2, beta-1,3-glucanase 2	-3.3099	0.5291	2.7808
AT3G28310	Hypothetical protein (DUF677)	-3.0674	0.2548	2.8126
AT1G58225	Uncharacterized protein	-2.9552	0.3915	2.5637
AT3G33166	Pseudo	-3.1865	1.5932	1.5932
AT3G04184	Uncharacterized protein	-3.1650	1.4934	1.6716
AT1G33200	Pseudo	-3.1444	1.5362	1.6081
AT2G44250	tRNA-splicing ligase, putative (DUF239)	-3.1368	1.6292	1.5076
AT2G41590	Ta11-like non-LTR retrotransposon	-2.8514	1.9314	0.9200
AT1G62240	Uncharacterized protein	-2.7851	0.7198	2.0653
AT5G05150	G18E, autophagy-related protein 18E	-2.6453	0.3794	2.2660
AT5G66053	Uncharacterized protein	-2.6657	2.2191	0.4466
AT1G11591	Uncharacterized protein	-2.8169	1.0852	1.7316
AT4G22490	Bifunctional inhibitor/lipid-transfer protein/seed storage 2S albumin superfamily protein	-2.7458	1.9373	0.8085
AT3G17265	F-box and associated interaction domains-containing protein	-2.7488	0.9288	1.8200
AT4G33355	Bifunctional inhibitor/lipid-transfer protein/seed storage 2S albumin superfamily protein	-2.7883	1.5421	1.2462
AT1G43010	Pentatricopeptide repeat (PPR) superfamily protein	-2.7258	1.8333	0.8924
AT1G05005	PPR containing protein	-2.7029	0.7930	1.9100
AT4G19430	Uncharacterized protein	-2.7415	1.7322	1.0093
AT5G63240	Carbohydrate-binding X8 domain superfamily protein	-2.7333	1.4796	1.2537
AT3G63095	Tetratricopeptide repeat (TPR)-like superfamily protein	-2.6985	1.2117	1.4868
AT5G19473	RPM1-interacting protein 4 (RIN4) family protein	-2.4968	0.4574	2.0394
AT2G47050	Plant invertase/pectin methylesterase inhibitor superfamily protein	-2.5991	1.1544	1.4447
AT4G06570	Pseudo	-2.5634	1.2817	1.2817
AT1G73065	Uncharacterized protein	-2.4075	1.9401	0.4674
AT1G72545	Uncharacterized protein	-2.4957	0.9000	1.5957
AT1G80625	Uncharacterized protein	-2.4245	1.5204	0.9042
AT2G22088	Uncharacterized protein	-2.3401	1.7933	0.5468
AT5G63375	Uncharacterized protein	-2.4206	1.1045	1.3161

AT3G12890	ASML2, activator of spomin::LUC2	-2.3257	0.6239	1.7018
AT5G26140	LOG9, Putative lysine decarboxylase family protein	-2.4044	1.1921	1.2123
AT1G43810	Uncharacterized protein	-2.3518	0.9112	1.4406
AT2G30540	Thioredoxin superfamily protein	-2.1209	0.1466	1.9743
AT5G42760	Leucine carboxyl methyltransferase	-2.3055	0.7154	1.5901
AT5G37950	UDP-Glycosyltransferase superfamily protein	-2.2155	1.7472	0.4683
AT3G11740	LURP-one-like protein (DUF567)	-2.2389	1.6942	0.5447
AT3G25573	Uncharacterized protein	-2.1922	1.7662	0.4260
AT4G08870	ARGAH2, Arginase/deacetylase superfamily protein	-2.3080	1.3715	0.9365
AT2G10280	Pseudo	-2.2780	1.3665	0.9114
AT5G22810	GDSL-like lipase/acylhydrolase superfamily protein	-2.1092	0.2841	1.8251
AT1G12064	Uncharacterized protein	-2.2613	1.3913	0.8700
AT1G23995	Transcription factor	-2.2149	0.6746	1.5403
AT2G24740	SDG21, SET domain group 21	-2.0757	0.2698	1.8059
AT1G03020	Thioredoxin superfamily protein	-1.9947	0.1177	1.8770
AT3G20950	CYP705A32, cytochrome P450, family 705, subfamily A, polypeptide 32	-2.1277	1.6650	0.4627
AT1G19830	SAUR-like auxin-responsive protein family	-2.2222	1.3340	0.8882
AT3G08560	VHA-E2, vacuolar H+-ATPase subunit E isoform 2	-2.0582	0.3351	1.7231
AT1G56270	RPB1a	-1.9111	0.0792	1.8319
AT5G37470	Hypothetical protein (DUF577)	-2.0597	1.5514	0.5083
AT2G31590	Uncharacterized protein	-2.1286	1.1953	0.9333
AT4G31950	CYP82C3, cytochrome P450, family 82, subfamily C, polypeptide 3	-2.0714	0.5928	1.4786
AT4G05091	Uncharacterized protein	-2.0985	0.7168	1.3817
AT5G67265	Uncharacterized protein	-1.9803	0.3142	1.6662
AT2G10820	Pseudo	-1.9772	0.3247	1.6525
AT5G08141	bZIP75, basic leucine-zipper 75	-2.0953	0.8114	1.2839
AT4G01180	XH/XS domain-containing protein	-2.0997	1.2111	0.8887
AT5G43695	Uncharacterized protein	-1.9860	0.4073	1.5787
AT2G17740	Cysteine/Histidine-rich C1 domain family protein	-2.0822	1.2588	0.8234
AT4G06477	Pseudo	-2.0871	0.9495	1.1376
AT2G18660	PNP-A, plant natriuretic peptide A	-1.8645	0.1152	1.7493
AT3G57960	Emsy N Terminus (ENT) domain-containing protein	-1.9733	0.4002	1.5731
AT4G26390	Pyruvate kinase family protein	-2.0398	0.7659	1.2739
AT3G17720	Pyridoxal phosphate (PLP)-dependent transferases superfamily protein	-1.9795	1.4708	0.5086

AT2G06810	Pseudo	-1.9545	0.4902	1.4642
AT5G18030	SAUR-like auxin-responsive protein family	-2.0128	1.0589	0.9539
AT4G25590	ADF7, actin depolymerizing factor 7	-1.8017	0.1493	1.6524
AT3G30730	Uncharacterized protein	-1.9656	0.6738	1.2918
AT4G16215	Uncharacterized protein	-1.9804	0.9337	1.0467
AT3G27895	Pseudo	-1.9738	1.1237	0.8502
AT4G34470	SK12, SKP1-like 12	-1.7780	0.1362	1.6418
AT1G62421	Uncharacterized protein	-1.8629	0.3941	1.4688
AT4G32375	Pectin lyase-like superfamily protein	-1.7613	0.1508	1.6105
AT2G07722	Uncharacterized protein	-1.9266	1.1914	0.7351
AT3G27415	Uncharacterized protein	-1.8539	0.4555	1.3983
AT4G28580	MGT5, magnesium transport 5	-1.9246	0.9623	0.9623
AT5G15430	Plant calmodulin-binding protein-like protein	-1.9212	0.9591	0.9621
AT2G04135	Pseudo	-1.9027	0.8580	1.0447
AT1G42150	Pseudo	-1.8975	0.9488	0.9488
AT4G23882	Heavy metal transport/detoxification superfamily protein	-1.8676	0.7355	1.1322
AT1G10455	B3 DNA-binding domain protein	-1.8210	0.5724	1.2486
AT5G35490	MRU1, mto 1 responding up 1	-1.8160	0.5773	1.2387
AT1G27890	Polynucleotidyl transferase, ribonuclease H-like superfamily protein	-1.8190	1.1902	0.6288
AT1G67670	Uncharacterized protein	-1.7919	1.2829	0.5090
AT4G18203	Uncharacterized protein	-1.8108	1.1023	0.7086
AT5G05040	Cystatin/monellin superfamily protein	-1.7264	1.3735	0.3529
AT5G52300	LTI65, CAP160 protein	-1.8092	1.0658	0.7434
AT2G28580	Transmembrane protein, putative (DUF247)	-1.7791	0.5699	1.2092
AT4G11540	Cysteine/Histidine-rich C1 domain family protein	-1.7044	0.3108	1.3936
AT2G23110	Late embryogenesis abundant protein, group 6	-1.7688	1.2311	0.5377
AT4G34210	SK11, SKP1-like 11	-1.8081	0.9461	0.8619
AT2G15750	Pseudo	-1.7760	0.6174	1.1586
AT5G44590	S-adenosyl-L-methionine-dependent methyltransferases superfamily protein	-1.7581	0.5362	1.2219
AT1G48940	ENODL6, early nodulin-like protein 6	-1.7720	0.6213	1.1507
AT2G07835	Uncharacterized protein	-1.7329	0.5113	1.2216
AT1G29780	Haloacid dehalogenase-like hydrolase (HAD) superfamily protein	-1.6672	0.2962	1.3709
AT1G36250	Pseudo	-1.6672	0.2962	1.3709

AT3G42386	Pseudo	-1.6672	0.2962	1.3709
AT2G36180	EF hand calcium-binding protein family	-1.7336	1.2002	0.5334
AT3G62640	DUF3511 domain protein (DUF3511)	-1.7640	0.8189	0.9451
AT5G16023	RTFL18, ROTUNDIFOLIA like 18	-1.7370	0.6657	1.0713
AT1G12340	Cornichon family protein	-1.7140	0.5413	1.1727
AT1G12663	Thionin-like protein	-1.6340	0.2796	1.3544
AT3G24510	Defensin-like (DEFL) family protein	-1.6591	0.3686	1.2906
AT2G07735	Pseudo	-1.7181	0.8253	0.8928
AT4G31441	Uncharacterized protein	-1.5977	0.2615	1.3362
AT5G38270	F-box family protein	-1.5977	0.2615	1.3362
AT2G15100	Pseudo	-1.5977	0.2615	1.3362
AT1G67160	F-box/kelch-repeat protein, putative (DUF295)	-1.5899	0.2576	1.3323
AT5G43360	PHT1;3, phosphate transporter 1;3	-1.6758	1.1144	0.5614
AT5G40900	Nucleotide-diphospho-sugar transferase family protein	-1.5859	0.2522	1.3336
AT2G16505	maternally expressed family protein	-1.6149	1.2670	0.3479
AT2G26630	Pseudo	-1.6995	0.8498	0.8498
AT3G13850	LBD22, LOB domain-containing protein 22	-1.6940	0.7921	0.9019
AT1G20680	Transport/golgi organization-like protein (DUF833)	-1.5751	0.3134	1.2617
AT3G09160	RNA-binding (RRM/RBD/RNP motifs) family protein	-1.6422	0.5763	1.0659
AT2G03610	F-box family protein	-1.6601	0.7332	0.9269
AT2G23660	LBD10, LOB domain-containing protein 10	-1.6096	0.4456	1.1640
AT5G59640	Pseudo	-1.5534	0.2977	1.2556
AT2G36770	UDP-Glycosyltransferase superfamily protein	-1.6329	0.6481	0.9848
AT3G51810	EM1, Stress induced protein	-1.6343	0.7591	0.8752
AT5G32481	Pseudo	-1.6336	0.8168	0.8168
AT1G23770	F-box family protein	-1.6310	0.8155	0.8155
AT1G52950	Nucleic acid-binding, OB-fold-like protein	-1.5937	1.0963	0.4974
AT1G51035	Uncharacterized protein	-1.5114	0.2474	1.2641
AT2G15940	Pseudo	-1.6211	0.8106	0.8106
AT4G09960	STK, K-box region and MADS-box transcription factor family protein	-1.4972	0.2108	1.2864
AT3G31935	Pseudo	-1.5546	0.4614	1.0932
AT2G07772	Uncharacterized protein	-1.5306	0.3929	1.1377
AT2G07682	Pseudo	-1.5464	0.9456	0.6008

Gene descriptions are according to the National Center for Biotechnology Information (NCBI, https://www.ncbi.nlm.nih.gov/).
